# On Depressed Vital Action

**Published:** 1818-06

**Authors:** 


*For the London Medical and Physical Journal.*

### *On Depressed Vital Action;* by Dr. Kinglake.

IT is in the power of accidental violence to the various functions of life so far to derange the vital condition of the animal œconomy, as to arrest the actions of living power, and induce a suspension of animation. This is more particularly liable to happen in violent injuries befalling the brain; by which, nervous energy is so shaken, as to be for a time inactive. A similar state may be also induced by sudden frights, by extreme anxiety, by excess of the various passions, and by whatever may occasion deliquium animi. The various terrors and vast exertions connected with warfare, and, indeed, the keen interest felt in arduous pursuits of any kind, may tend to depress the action of vital power, in a degree that might endanger the extinction of life, were the fallen state, by any mismanagement, to be neglected and increased. In concussions of the brain, from external violence, the vital action is often so far suspended, as to render the motion of the heart and arteries imperceptible. This is an awful depression of nervous energy,—a pause in animation, full of danger, and demanding appropriate treatment, to obviate positive death. Whatever cause may have had the serious effect of arresting vital action, it deserves to be carefully considered, that the natural disposition to its renewal should be in every way promoted, not repressed.

In concussions of the brain, the usual influence of the nervous power is not exerted; and, though the power is not destroyed, its comparative inaction is very analogous to the state of death. It is evident, that, in these circumstances of vital embarrassment, in which the usual stimulant powers have lost their customary effect, it would be right to endeavour to awaken the latent energies of life; and that this should be attempted by stimulating the stomach, if swallowing be practicable, by irritating the nostrils, by inflating the lungs, by applying increased temperature to the region of the heart, and by general friction. If these means should avail in renovating the action of the heart and arteries, life will not only be restored, but the force with which the restoration will obtain, may lead to immoderate excitement, requiring the abstraction of blood to allay its violence. This is especially the case in injuries of the brain from external violence, going the length of momentarily extinguishing the active powers of life. It is not often, that, on the receipt of mischief of that nature, the arterial action immediately ceases; but, generally, it becomes extremely languid and fluttering.

This is the critical state demanding appropriate treatment. If the popular remedy of bleeding be resorted to, whilst the vital action is declining from the hurtful violence inflicted, and before an effort at re-action has been made, irretrievable destruction will be the probable consequence. It is therefore of an importance equal to the value of life itself, not to be too precipitate on these occasions. Let the action of the heart and arteries be firmly established before blood be drawn, for the purpose of obviating the evils of excessive action. Bleeding, on these occasions, is supposed, by diminishing the contents of the sanguiferous vessels, at once to excite them to contraction, and to lessen the stimulus of distention,—thus assisting the circulation, and preventing the injurious effects of vascular tension. These indications of cure are sufficiently rational, when they can be carried into effect; but where is the prospect of their being accomplished during the existence of a state approaching to vascular inaction? If the contractile power of the vessel cannot be excited by its distended state, will it be more strongly acted on, by removing that natural stimulus by bleeding? This would be opposing what was intended to be effected, and incurring the very mischief designed to be avoided. Experience has sufficiently ascertained the correctness of this reasoning, and high practical authorities have sanctioned the utility of the distinction here proposed. In cases of rigor, extreme cold, terrific surprise, syncope, and in all the different forms and degrees of asphyxia, the sudden abstraction of stimulant agency induces the dormant state of life, and it will be necessary to restore this exciting influence, to afford a chance for the renewal of vital action. Either the innate tendency to revived exertion, or the agency of exciting means, should precede the popular custom of endeavouring to recall the fallen energy, by the removal of blood, in the fallacious expectation that a discharge of that fluid would induce a contractile effort in the heart and arteries, and would thereby advance and finish the work of resuscitation.

Some judicious remarks were some time since published in your Journal, respecting the fatal effects of precipitating exhausted patients, after the sanguinary conflicts of battles, to surgical operations. It cannot be doubted, that the additional shock, given to an almost expiring state of debility, from the various exhausting influence of a destructive engagement, must necessarily be most hurtful, and, in numerous instances, must have proved mortal. When life is overwhelmed, and precariously ebbing, whether by a deranged or exhausted state of vital power, it is equally and imperiously necessary, to admit of a recruiting period, in which the tottering condition may receive some prop against the demolition with which it is threatened. This is the interval that is held to be indispensably necessary for the regeneration of lost power, before measures are taken to obviate the injuries that might accrue from an eventual excess of re-action. If this caution be observed, assistance will not be unseasonably obtruded, but afforded at the time when it will avail in obviating ulterior mischief, and in forwarding the desired recovery. The occasions for medical aid in cases approaching, or actually amounting, to suspended animation, and indispensably requiring appropriate relief, are numerous; it is therefore of the utmost consequence, that an attempt to assist should be made on principles that may be at once sanctioned by experience and reason. The object is, to recall into action the sinking power of life: this will be best accomplished by temporarily sustaining the feeble struggle by suitable excitement, until it shall have attained a degree of exertion that would leave nothing to dread on the score of natural capability to renovate, in sufficient force, the active energies of life. When this shall have been effected, it remains, as is usual in other instances of active disease, so to regulate the re-active exertions as to insure them a salutary course, and, consequently, a favourable termination.

*Taunton; March* 1, 1818.

